# Riluzole regulates pancreatic cancer cell metabolism by suppressing the Wnt-β-catenin pathway

**DOI:** 10.1038/s41598-022-13472-y

**Published:** 2022-06-30

**Authors:** Sanjit K. Roy, Yiming Ma, Bao Q. Lam, Anju Shrivastava, Sudesh Srivastav, Sharmila Shankar, Rakesh K. Srivastava

**Affiliations:** 1 Stanley S. Scott Cancer Center, School of Medicine, Louisiana State University Health-New Orleans, New Orleans, LA 70122 USA; 2 Kansas City VA Medical Center, Kansas City, MO 66128 USA; 3 St. Joseph’s Hospital and Medical Center, Phoenix, AZ 85013 USA; 4 Department of Biostatistics and Data Science, School of Public Health and Tropical Medicine, Tulane University School of Medicine, New Orleans, LA 70122 USA; 5 Department of Genetics, Louisiana State University Health Sciences Center, New Orleans, LA USA; 6 Southeast Louisiana Veterans Health Care System, New Orleans, LA 70112 USA

**Keywords:** Biochemistry, Cancer, Gastroenterology, Oncology

## Abstract

Most cancer cells rely on aerobic glycolysis to support uncontrolled proliferation and evade apoptosis. However, pancreatic cancer cells switch to glutamine metabolism to survive under hypoxic conditions. Activation of the Wnt/β-catenin pathway induces aerobic glycolysis by activating enzymes required for glucose metabolism and regulating the expression of glutamate transporter and glutamine synthetase. The results demonstrate that riluzole inhibits pancreatic cancer cell growth and has no effect on human pancreatic normal ductal epithelial cells. RNA-seq experiments identified the involvement of Wnt and metabolic pathways by riluzole. Inhibition of Wnt-β-catenin/TCF-LEF pathway by riluzole suppresses the expression of PDK, MCT1, cMyc, AXIN, and CyclinD1. Riluzole inhibits glucose transporter 2 expression, glucose uptake, lactate dehydrogenase A expression, and NAD + level. Furthermore, riluzole inhibits glutamate release and glutathione levels, and elevates reactive oxygen species. Riluzole disrupts mitochondrial homeostasis by inhibiting Bcl-2 and upregulating Bax expression, resulting in a drop of mitochondrial membrane potential. Finally, riluzole inhibits pancreatic cancer growth in KPC (Pdx1-Cre, LSL-Trp53^R172H^, and LSL-Kras^G12D^) mice. In conclusion, riluzole can inhibit pancreatic cancer growth by regulating glucose and glutamine metabolisms and can be used to treat pancreatic cancer.

## Introduction

The incidence of pancreatic cancer is expected to rise significantly in near future. According to the American Cancer Society, pancreatic cancer will be the number one cause of cancer-related death by 2050. Pancreatic ductal adenocarcinoma (PDAC) accounts for 95% of pancreatic cancers; after diagnosis, the 5-year survival rate of the PDAC is less than 10%^1^. It is characterized by silent growth, late detection, poor prognosis, and resistance to chemotherapy and radiation^2,3^. Since most PDAC are detected at an advanced stage, many pancreatic cancers are not resectable because cancer cells are already metastasized to various organs^4^. The first-line regimens (FOLFIRINOX and gemcitabine/nab-paclitaxel) for advanced pancreatic cancer patients provide limited survival benefits and are associated with enormous toxicities^5–7^. Therefore, there is an unmet need to develop an effective and non-toxic drug for the treatment of pancreatic cancer.

Dysregulated cellular metabolism is the key feature of cancers^8^. Both hypoxia and oncogenic mutations have been shown to rewire tumor metabolism. Cancer cells with dysregulated metabolism demonstrate higher rates of glucose uptake than normal tissue and favor aerobic glycolysis^9,10^. In addition to the dependency on glycolysis, cancer cells are addicted to increased rates of glutamine metabolism^11,12^. Although the requirement for mitochondrial ATP production is significantly decreased in glycolytic cancer cells, the demand for Krebs cycle (Citric Acid Cycle or TCA)-derived biosynthetic precursors and nicotinamide adenine dinucleotide phosphate is unchanged or even increased^13^. Cancer cells generally depend on elevated glutaminolysis to maintain a functional Krebs cycle and compensate for these changes.

Glutamate dehydrogenase 1 (GDH1) transmits its signals through antioxidant glutathione peroxidase 1 (GPx-1) to regulate redox homeostasis, malignant cell proliferation and tumor growth^14^. GDH1 plays a crucial role in redox homeostasis of cancer cells by controlling the intracellular levels of its product alpha-ketoglutarate (α-KG)) and subsequent metabolite fumarate. Fumarate binds to and activates the enzyme GPx-1. Inhibition of GDH1 causes imbalanced redox homeostasis, resulting in suppression of cancer cell proliferation and tumor growth^14^. Furthermore, targeting mitochondrial glutaminase activity inhibits oncogenic transformation^15^.

The enhanced glycolysis causes increased lactate secretion in the tumor microenvironment resulting in extracellular acidification. During hypoxia, hypoxia-inducible factor (HIF) regulates the expression of lactate dehydrogenase A (LDHA) and monocarboxylate transporter 4 (MCT4), which leads to the secretion of lactate from cells. Increased activity of LDHA in tumor cells results in upregulation of NADH relative to NAD^+^. Because cancer cells exhibit a high glycolytic rate, there is an enhanced shuttling of the glycolytic intermediates to the pentose phosphate pathway resulting in augmented NADPH production that impedes oxidative stress. The ratio of NAD(P)H to NAD(P)^+^ determines ATP production. While normal differentiated cells primarily rely on mitochondrial oxidative phosphorylation to generate energy, most cancer cells rely on aerobic glycolysis, a phenomenon termed “the Warburg effect”. Thus, the induction of hypoxia facilitates a major metabolic shift in cancer cells. Certain cancer-associated mutations enable cancer cells to acquire and metabolize nutrients in a manner which facilitates proliferation rather than efficient ATP production.

Riluzole is an FDA approved drug for treating amyotrophic lateral sclerosis (ALS) and clinical trials are being carried out for several human diseases^16–18^. Riluzole inhibits glutamate release through inactivation of voltage-dependent ion channels^19–21^, and regulates signal transduction pathway through glutamate receptors^21^. Clinical studies have demonstrated the use of riluzole as antidepressant and anti-anxiety drugs^22–26^. Despite these studies, the molecular mechanisms by which riluzole exerts its anticancer activities in pancreatic cancer have never been examined.

Wnt pathway plays a major role in pancreatic cancer development^27,28^. In the absence of Wnt ligands, the destruction complex (GSK3, CK1α, axin, and APC) promotes the phosphorylation of β-catenin, resulting in its ubiquitylation, and subsequent degradation^29–31^. The binding of Wnt ligands with Frizzled (Fzd) receptors and the Wnt co-receptor LRP5 or LRP6 activates the Dishevelled (Dvl) cytoplasmic phospho protein, which inhibits β-catenin phosphorylation and degradation. The translocation of β-catenin into the nucleus causes binding to TCF/LEF proteins, and acts as a transcriptional co-activator to modulate the expression of target genes such as Cyclin D1, cyclooxygenase 2 (COX-2), pyruvate dehydrogenase kinase (PDK), monocarboxylate lactate receptor 1 (MCT-1), and Myc^32–35^. There are no studies examining the effects of riluzole on metabolism through Wnt signaling pathway in pancreatic cancer.

The purpose of this study was to examine the molecular mechanisms by which riluzole regulates pancreatic cancer cell metabolism. Our data demonstrate that riluzole inhibits Wnt-β-catenin/TCF-LEF pathway and suppresses the expression of PDK, MCT1, cMyc, AXIN, Bcl-2, and CyclinD1. Riluzole inhibits glucose transporter 2 expression, glucose uptake, lactate dehydrogenase A expression, and NAD + level. Furthermore, riluzole inhibits glutamate release, and glutathione level, and elevates ROS. Riluzole disrupts mitochondrial homeostasis by inhibiting Bcl-2 and upregulating Bax expression. Finally, riluzole inhibits pancreatic cancer growth and development in KPC mice. In conclusion, riluzole can inhibit pancreatic cancer growth by regulating glucose and glutamine metabolisms and can be used for the treatment of pancreatic cancer.

## Materials and methods

### Reagents

JC-1 mitochondrial membrane potential assay kit, and Pierce BCA Protein Assay Kit were purchased from Thermo Fisher (Grand Island, NY). BD Matrigel was purchased from BD Bioscience (San Jose, CA). TRIzol and polybrene were purchased from Invitrogen (Grand Island, NY). CellTiter-Glo® Luminescent Cell Viability Assay and Luciferase assay kits were purchased from Promega Corporation (Madison, WI). All other chemicals were purchased from Sigma-Aldrich (St. Louis, MO).

### Cell culture

Human pancreatic cancer cell lines PANC-1, AsPC-1, Hs 766 T and MIA PaCa-2 were purchased from American Type Culture Collection (ATCC), Manassas, VA, and were authenticated by the vendor using short tandem repeat (STR) profiling. Cell lines were grown in Dulbecco's Modified Eagle's Medium (DMEM) with 10% Fetal Bovine Serum (HyClone) with antibiotics. Human pancreatic cancer stem cells (CSCs, CD24 + /CD44 + /CD133 +) were isolated from primary tumors and grown in stem cell culture medium as per the supplier’s instructions (obtained from Celprogen, Torrance, CA). Human pancreatic normal ductal epithelial (HPNE) cells were purchased from ATCC. All cells were *Mycoplasma* free (as per detection kit from Lonza) and used within 6 months of continuous passage.

### Lentiviral particle production and transduction

The protocol for lentivirus production and transduction was described elsewhere^36,37^. Briefly, 293 T cells were transfected with 4 µg of plasmid and 4 µg of the lentiviral vectors using Lipofectamine-3000 as per protocol from Invitrogen. After collecting supernatant, PEG-it virus precipitation solution (SBI System Biosciences) was added, and viral particles were collected through ultracentrifugation. CSCs and cancer cell lines were transduced with lentiviral particles with 6 μg/ml polybrene^38,39^.

### Apoptosis

Pancreatic cancer cells were treated with or without riluzole for various time points. Apoptosis was measured by TUNEL (terminal deoxynucleotidyl transferase (TdT)-mediated dUTP nick end labelling) assay as per the manufacturer’s protocol (Life Technologies, Grand Island, NY).

### Cell cycle analysis

Cell cycle analysis was performed as we described elsewhere^40^. In brief, cells (5 × 10^5^) were seeded in cell culture dishes. After 24 h, the medium was removed and replaced with fresh medium containing riluzole 0–20 µM for 48 h. Cell cycle analysis was performed by measuring the amount of propidium iodide (PI) in ethanol fixed cells. In brief, cells were harvested by trypsinization and fixed with cold 70% ethanol for 24 h. Cells were rinsed 3 times with ice-cold PBS and resuspended in 1 ml of permeabilizing solution (Triton X 100 (0.25%), sodium azide (0.01%) and RNAs A (100 μg/μl Sigma-Aldrich) in PBS for 10 min. Cells were rinsed once with PBS, resuspended with 1 ml of PBS with PI (2.5 mg/ml) and incubated for 15 min at 4 °C. Cell cycle analysis was performed using a flow cytometer (Becton Dickinson).

### Cell viability

The CellTiter-Glo® Luminescent Cell Viability Assay is a homogeneous method of determining the number of viable cells in culture based on quantitation of the ATP present, an indicator of metabolically active cells. Cells were seeded in 96-well plate and treated with or without riluzole for various time points. Cell viability was measured by CellTiter-Glo® assay as per manufacturer’s protocol (Promega Corporation, Madison, WI).

### Colony formation assay

Colony formation assays were performed as described elsewhere^37,41^. In brief, pancreatic cancer cells were seeded at a low density into 6-well plates for about 3 weeks. Following incubation, colonies were fixed with methanol, stained with 0.5% crystal violet and counted under a microscope.

### Measurement of mitochondrial membrane potential

Cells were seeded in a 96-well plate and treated with or without riluzole for various time points. Mitochondrial membrane potential was measured by MitoProbe JC-1 Assay Kit as per manufacturer’s protocol (Thermo Fisher Scientific).

### Western blot analysis

The Western blot analysis was performed as we described elsewhere^37,42^. In brief, cells were washed once with ice-cold PBS and suspended in RIPA lysis buffer (10 mM Tris–HCl, pH 7.4, 140 mM NaCl, 1% Triton X-100, 1% Sodium deoxycholate, 0.1% SDS, 1 mM Na_3_VO_4_, 1 mM NaF, and 1 mM EDTA) added with a cocktail of protease inhibitors (Sigma-Aldrich, St. Louis, MO). Lysis was performed on ice for 30 min, and the samples were centrifuged at 17,000 × g for 30 min. The bicinchoninic acid (BCA) Protein Assay Kit was used to measure the concentration of crude protein (Thermo Fisher Scientific, Rockford, IL). Equal amounts of crude protein lysates were loaded and separated by SDS-PAGE, and gels were blotted to polyvinylidene difluoride membranes. After transfer of proteins, membranes were incubated with primary antibodies, washed, and incubated with peroxidase-conjugated secondary antibodies. Finally, protein blots were developed with an enhanced chemiluminescent detection kit and visualised with an imaging system.

### TCF/LEF reporter assay

Lentiviral particles expressing cop-GFP and luciferase genes (TCF/LEF-mCMV-EF1-Neo) were prepared as described elsewhere^43^. Cells were transduced with lentiviral particles containing gene of interest. Transduced cells (5–10,000 cells per well) were seeded in 96-well plates and treated with or without riluzole for various time points. At the end of incubation period, luciferase reporter activity was measured as per the manufacturer's instructions (Promega Corp., Madison, WI).

### Glucose uptake

Cells were labelled with 2.5 µg/ml of 2-deoxy-2-[(7-nitro-2,1,3-benzoxadiazol-4-yl)amino]-D-glucose (2-NBD Glucose) for 30 min, and treated with riluzole for 24 h. Cells were washed and resuspended in PBS. Fluorescence was measured (excitation 544 nm and emission at 590 nm).

### Measurement of NAD + 

Pancreatic cancer cells were seeded in 24-well plate and treated with riluzole (0–10 µM) for 1 h and total cellular NAD + concentration was measured at 450 nm by a NAD/NADH assay kit (Cayman).

### Glutamate release assay

Pancreatic cancer cells and CSCs were treated with riluzole (0–10 µM) for 24 h. Glutamate release was measured by the Amplex® Red Glutamic Acid/Glutamate Oxidase Assay Kit with a fluorometer using excitation at 540 nm and emission at 590 nm (Invitrogen).

### Measurement of intracellular GSH

Pancreatic cancer cells and CSCs were treated with riluzole (0–10 µM) for 24 h. Intracellular total GSH was detected by measuring the product of glutathionylated DTNB at 405 nm (GSH assay kit, Cayman Chemical).

### Measurement of reactive oxygen species

Cells were pre-treated with NAC (3 mM) for 2 h, followed by treatment with riluzole (0–10 µM) for 24 h. Cells were labeled with 2’,7’-dichlorofluorescein diacetate (DCFDA / H2DCFDA) and ROS production was measured with a fluorometer using excitation at 495 nm and emission at 529 nm (Cellular Reactive Oxygen Species Detection Assay Kit, Abcam).

### Quantitative real-time PCR

Quantitative real-time PCR was performed as we described elsewhere^37^. In brief, total RNA was first extracted with TRIzol reagent (Invitrogen), and the cDNA was generated by the Reverse Transcription System (Promega) in a 20 μl reaction containing 1 μg of total RNA. A 0.5 μl aliquot of cDNA was amplified by Fast SYBR Green PCR Master Mix (Applied Biosystems) in each 20 μl reaction. PCR reactions were run on the ABI 7900 Fast Real-Time PCR system (Applied Biosystems).

The following gene-specific primers were used:

$$\begin{array}{*{20}l} {\beta - {\text{Catenin }}\left( {5^{\prime} - {\text{TGAGGACAAGCCACAAGATTAC}} - 3^{\prime}, \, 5^{\prime} - {\text{TCCACCAGAGTGAAAAGAACG}} - 3^{\prime}} \right)} \hfill \\ {{\text{Wnt}}3a \, (5^{\prime} - GACTATCCTGGACCACATGC - 3^{\prime}, \, 5^{\prime} - {\text{GGACTCACGGTGCTTCTCTA}} - 3^{\prime})} \hfill \\ {{\text{Wnt}}5a \, \left( {5^{\prime} - {\text{TAGCAGCATCAGTCCACAAA}} - 3^{\prime}, \, 5^{\prime} - {\text{CAAAACACGGCATCTCTCTT}} - 3^{\prime}} \right)} \hfill \\ {{\text{WSP}}1 \, (5^{\prime} - {\text{ TTC TGT GCC AGA CAT TGC TC }} - 3^{\prime}, \, 5^{\prime} - {\text{ CCA ACT GGG TGA CTC TGG TT }} - 3^{\prime})} \hfill \\ {{\text{Cyclin }}D1 \, (5^{\prime} - {\text{TTCAAATGTGTGCAGAAG GA }} - 3^{\prime}, \, 5^{\prime} - {\text{GGGATGGTCTCCTTCATC TT }} - 3^{\prime})} \hfill \\ {{\text{Axin}}1 \, (5^{\prime} - {\text{ CTG CCG ACC TTA AAT GAA GA }} - 3^{\prime}, \, 5^{\prime} - {\text{ AAC TCT CTG CCT TCG CTG TA }} - 3^{\prime})} \hfill \\ {{\text{GSK}}3\beta \, (5^{\prime} - {\text{ ATT AAA GCTCACCCCTGG AC }} - 3^{\prime}, \, 5^{\prime} - {\text{TCACCA GCA CTG AAGTTGAA}} - 3^{\prime})} \hfill \\ {{\text{PDK}}1(5^{\prime} - {\text{ ATA CGG ATC AGA AAC CGA CA }} - 3^{\prime}, \, 5^{\prime} - {\text{CAG ACG CCT AGC ATT TTC AT}} - 3^{\prime})} \hfill \\ {{\text{LDHA }}(5^{\prime} - {\text{ AGGCTACACATC CTGGGCTA }} - 3^{\prime}, \, 5^{\prime} - {\text{CCCAAAATGCAAGGA ACA CT}} - 3^{\prime})} \hfill \\ {{\text{MCT}}1 \, (5^{\prime} - {\text{ TCC AGC TCT GAC CAT GAT TG }} - 3^{\prime},5^{\prime} - {\text{ GCC CCC AAG AAT TAG AAA GC }} - 3^{\prime})} \hfill \\ {{\text{Bcl}} - 2 \, \left( {5^{\prime} - {\text{AGATGGGAACACTGGTGGAG}} - \, 3^{\prime}, \, 5^{\prime} - {\text{CTTCCCCAAAAGAAATGCAA}} - \, 3^{\prime}} \right)} \hfill \\ {{\text{Bax }}(5^{\prime} - {\text{GCTGGACATTGGACTTCCTC}} - 3^{\prime} \, 5^{\prime} - {\text{CTCAGCCCATCTTCTTCCAG}} - 3^{\prime})} \hfill \\ {{\text{Glut}}2 \, (5^{\prime} - {\text{ TCG TCT CCT TTG ACA TTT CC}} - 3^{\prime}, 5^{\prime} - {\text{ CCA GTT GGT GGA GAA AAC AG }} - 3^{\prime})} \hfill \\ {{\text{Myc }}(5^{\prime} - {\text{CGA CGA GAC CTT CAT CAA AA}} - 3^{\prime}, \, 5^{\prime} - {\text{TGC TGT CGT TGA GAG GGT AG}} - 3^{\prime}} \hfill \\ {{\text{HK}} - {\text{GAPD }}\left( {5^{\prime} - {\text{GAG TCA ACG GAT TTG GTC GT}} - 3^{\prime}, \, 5^{\prime} - {\text{TTG ATT TTG GAG GGA TCT CG}} - 3^{\prime}} \right)} \hfill \\ \end{array}$$

### RNA-Seq

RNA was extracted from control and riluzole-treated cells with the RNeasy Kit and on column DNA digestion (Qiagen). For RNA-seq of pancreatic cancer cells, polyA mRNA was isolated and libraries were prepared using the TrueSeq Standard mRNA Kit according to the manufacture’s protocol (Illumina). After library preparations, samples were sequenced on a NextSeq 500 instrument with single-end 75 bp reads to a depth of 30 to 50 M reads/sample.

### KPC (Pdx1-Cre, LSL-Trp53^R172H^, and LSL-Kras^G12D^) mice

This study was approved by the Institutional Animal Care and Use Committee (IACUC) of the LSU Health Sciences Centre, New Orleans. All experimental procedures also followed the relevant guidelines and regulations according to state, national and international standards for ethics in animal experimentation. Additionally, this study is compliant with the ARRIVE guidelines. KPC (Pdx1-Cre, LSL-Trp53^R172H^, and LSL-Kras^G^^12^^D^) mice were generated as described elsewhere^44^. KPC mice (about 6 weeks old, n = 7) were injected ip with or without riluzole (20 mg/kg, Monday through Friday) for about 12 weeks^44^. At the end of the treatment, mice were euthanized by CO2 inhalation followed by thoracotomy. Histological examination of the pancreas was performed by H&E staining. Numbers of PanINs and PDAC were quantified as described elsewhere^45^. Animals were kept in pathogen-free conditions, at 21 to 25 °C and exposed to a normal diurnal variation under 12 h of light and 12 h of dark with food and water available ad libitum. Mice were weighed and observed regularly for signs of distress.

### Quantification and statistical analysis

GSEA v2.1.0 GraphPad PRISM 7, R 3.2.3 and Python 2.7.2 software packages were used to perform the statistical analyses. Statistical differences between groups were analyzed using the Student t test or Analysis of Variance (ANOVA). The threshold for statistical significance is *p* < 0.05, unless otherwise stated. The mean ± SD or SEM was calculated for each experimental group, unless otherwise specified.

## Results

### Riluzole inhibited cell viability and colony formation, and induced apoptosis in pancreatic cancer cell lines and CSCs while sparing human normal pancreatic ductal epithelial cells

**Figure 1:**
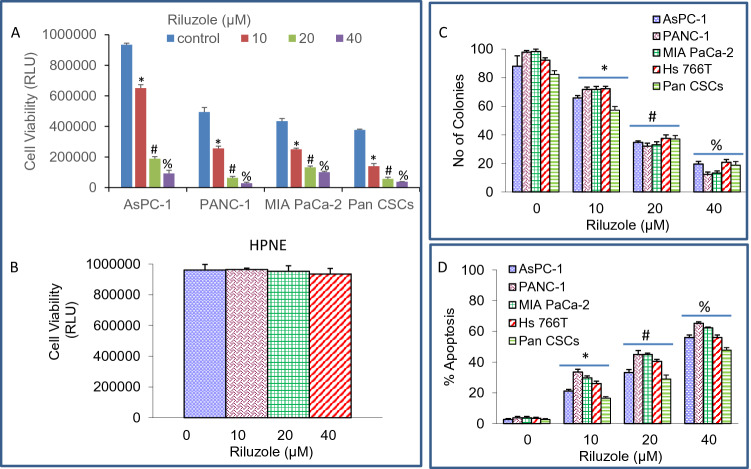
Inhibition of cell viability and colony formation, and induction of apoptosis by riluzole. (**A**,**B**), Pancreatic cancer cell lines (AsPC-1, PANC-1, and MIA PaCa-2), pancreatic cancer stem cells (Pan CSCs), and human normal pancreatic ductal epithelial (HPNE) cells were seeded in 96-well plates, treated with or without riluzole (0–40 μM) for 72 h and cell viability was measured by CellTiter-Glo Luminescent Cell Viability Assay (Promega). (**C**), Pancreatic cancer cell lines (AsPC-1, PANC-1, MIA PaCa-2 and Hs 766 T) and Pan CSCs were seeded in dishes and treated with riluzole (0–40 μM). Number of colonies formed at 21 days were counted as described in Material and Methods. (**D**), Pancreatic cancer cell lines (AsPC-1, PANC-1, MIA PaCa-2 and Hs 766 T) and CSCs were seeded and treated with riluzole (0–40 µM) for 72 h. Apoptosis was measured by TUNEL assay. Data represent mean ± SD (n = 4). *, #, and % = significantly different from control and each other; *p* < 0.05.

We first examined the effects of riluzole on pancreatic cancer cells and CSCs. Pancreatic CSCs (CD133^+^, CD24^+^, CD44^+^, and ESA^+^) were isolated from primary tumors and characterized as we described earlier^46^. Riluzole inhibited cell viability and colony formation in pancreatic cancer cell lines (AsPC-1, PANC-1, MIA PaCa-2) and CSCs (Fig. 1 A). By comparison, riluzole had no effect on the viability of human pancreatic normal ductal epithelial (HPNE) cells (Fig. 1B). Riluzole inhibited colony formation in pancreatic cancer cell lines and CSCs (Fig. 1C). Riluzole induced apoptosis in pancreatic cancer cell lines and CSCs (Fig. 1D). Maximum induction of apoptosis was seen in PANC-1 cells, whereas Pan CSCs were least responsive. These data suggest that riluzole can be used for the treatment of pancreatic cancer.

### Riluzole regulated Wnt/β-catenin/TCF-LEF pathway and mitochondrial proteins

**Figure 2:**
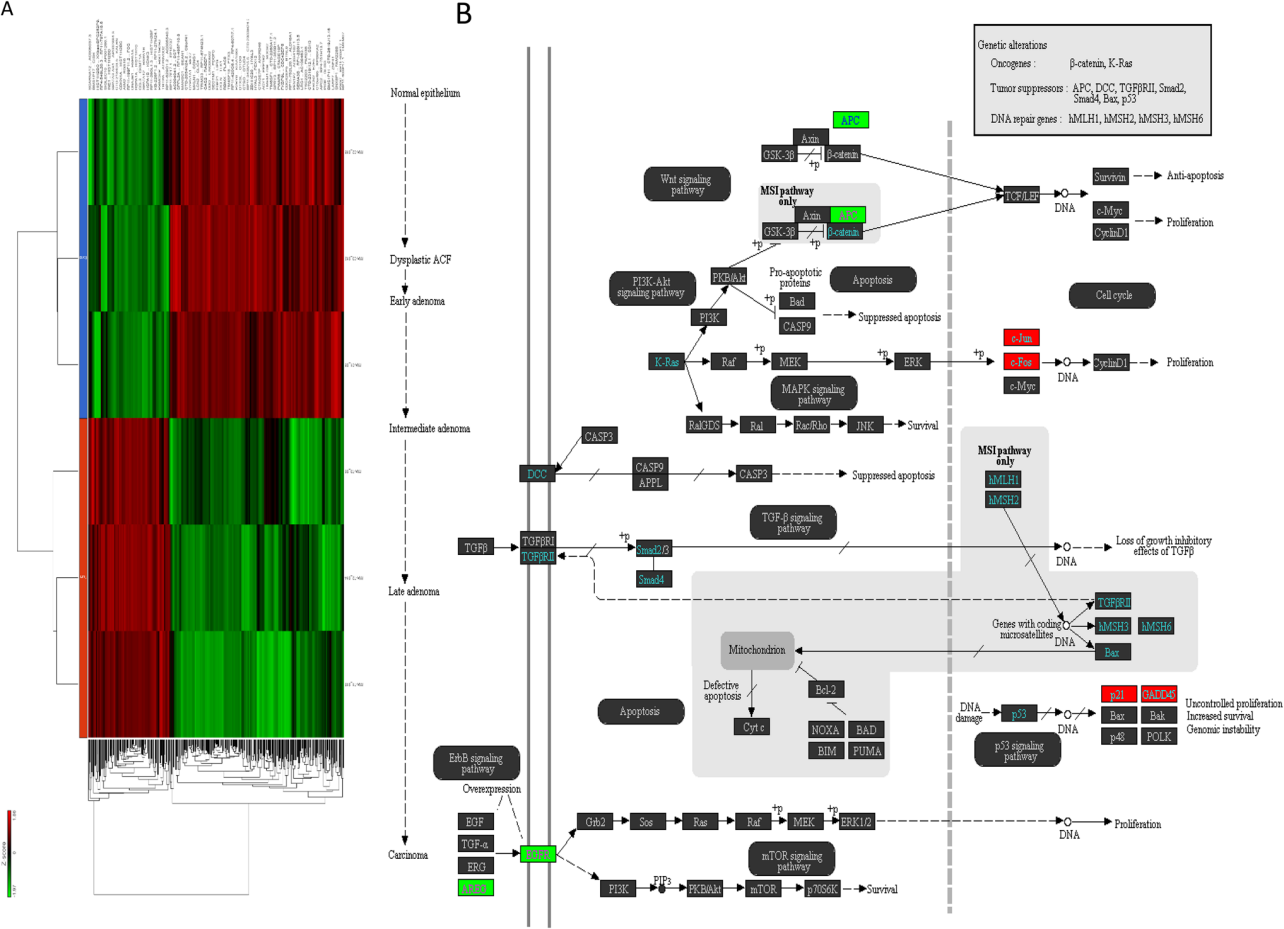
Riluzole regulates Wnt-β-catenin and mitochondrial pathways by RNA-Seq. Pancreatic cancer cells (MIA PaCa-2) were treated with Riluzole for 36 h. RNA was isolated. RNA-Seq was performed as described in Materials and methods. (**A**), Heat Map. Differential expression of genes in MIA PaCa-2 cells treated with or without riluzole. (**B**), Data analysis identified modulation of Wnt-β-catenin and mitochondrial pathways by riluzole in pancreatic cancer cells.

We next perform RNA-Seq experiment to identify pathways which regulate cell metabolism and mitochondrial functions in response to treatment with riluzole. As shown in Fig. 2A, gene expression pattern was different in control and riluzole-treated cells. Riluzole mainly regulated Wnt/β-catenin/TCF-LEF, MAP kinase, TNF and mitochondrial pathways (Fig. 2B). Since our focus was to examine the effects of riluzole on cancer cell metabolism, we selected to examine the regulation of Wnt/β-catenin/TCF-LEF pathway which controls the expression of several genes required for glucose and glutamine metabolisms, and mitochondrial function.

### Riluzole inhibited components of β-catenin/TCF-LEF pathway

**Figure 3:**
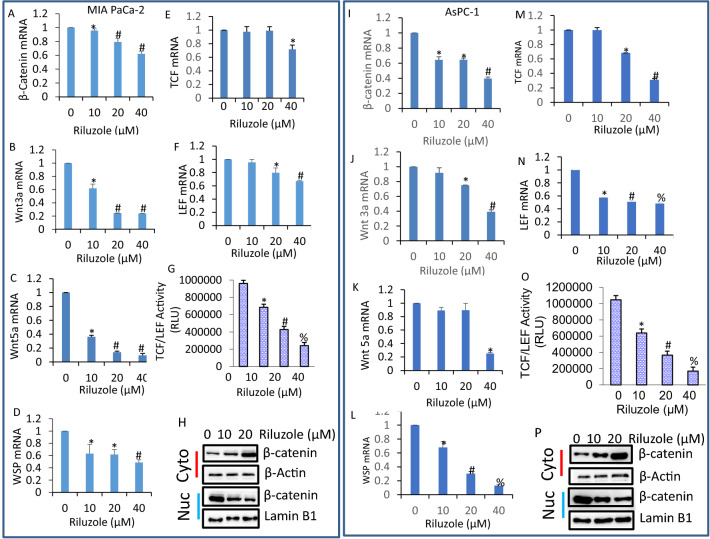
Inhibition of β-catenin/TCF-LEF1 pathway by riluzole. (**A**–**F**), Pancreatic cancer MIA PaCa-2 cells were treated with riluzole (0–40 µM) for 36 h. RNA was extracted and the expression of β-catenin, Wnt3a, Wnt5a, WSP, TCF and LEF was measured by q-RT-PCR. Data represent mean ± SD (n = 4). *, and # = significantly different from control and each other; *p* < 0.05. (**G**), MIA PaCa-2 cells were stably transduced with TCF/LEF1-responsive GFP/firefly luciferase viral particles (pGreen Fire1-TCF/LEF1 with EF1, System Biosciences). After transduction, cells were treated with riluzole (0–40 µM) for 36 h. TCF/LEF1 reporter activity was measured by luciferase assay as we described^73^. Data represent mean ± SD (n = 4). *, # and % = significantly different from control and each other; *p* < 0.05. (**H**), MIA PaCa-2 cells were treated with riluzole (0–40 µM) for 36 h. Cells were harvested and cytoplasmic and nuclear fractions were prepared. The expression of β-catenin in the cytoplasmic and nuclear fractions was measured by the Western blot analysis. β-Actin and Lamin B1 were used as loading controls in cytoplasmic and nuclear fractions, respectively. (**I–N**), Pancreatic cancer AsPC-1 cells were treated with riluzole (0–40 µM) for 36 h. RNA was extracted and the expression of β-catenin, Wnt3a, Wnt5a, WSP, TCF and LEF was measured by q-RT-PCR. Data represent mean ± SD (n = 4). *, # and % = significantly different from control and each other; *p* < 0.05. (**O**), AsPC-1 cells were stably transduced with TCF/LEF1-responsive GFP/firefly luciferase viral particles (pGreen Fire1-TCF/LEF1 with EF1, System Biosciences). After transduction, cells were treated with riluzole (0–40 µM) for 36 h. TCF/LEF1 reporter activity was measured by luciferase assay as we described^73^. Data represent mean ± SD (n = 4). *, # and % = significantly different from control and each other; *p* < 0.05. (**P**), AsPC-1 cells were treated with riluzole (0–40 µM) for 36 h. Cells were harvested and cytoplasmic and nuclear fractions were prepared. The expression of β-catenin in the cytoplasmic and nuclear fractions was measured by the Western blot analysis. β-Actin and Lamin B1 were used as loading controls in cytoplasmic and nuclear fractions, respectively.

Since our RNA-Seq experiments identified the regulation of β-catenin/TCF-LEF1 pathway by riluzole in pancreatic cancer, we examined the effects of riluzole on the expression of components of this pathway by q-RT-PCR and TCF-LEF1 transcriptional activity by luciferase assay (Fig. 3). Riluzole inhibited the expression of β-catenin, Wnt3a, Wnt5a, WSP, TCF and LEF in both MIA PaCa-2 and AsPC-1 pancreatic cancer cells (Fig. 3A–F and I–N). Since riluzole inhibited the expression of genes in β-catenin pathway, we next sought to measure the TCF-LEF1 transcriptional activity which is regulated by β-catenin. Riluzole inhibited TCF-LEF1 transcriptional activity in both MIA PaCa-2 and AsPC-1 cells (Fig. 3G,O). Upon activation of Wnt pathway, β-catenin is translocated to the nucleus and induces gene transcription. MIA PaCa-2 and AsPC-1 cells were treated with riluzole, cells were harvested and the expression of β-catenin in the cytoplasmic and nuclear fractions was measured by the Western blot analysis. Untreated pancreatic cancer cells expressed high levels of β-catenin in the nucleus compared to that in cytoplasm (Fig. 3H,P). Riluzole inhibited the translocation of β-catenin to the nucleus in MIA PaCa-2 and AsPC-1 cells. These data suggest that riluzole can inhibit pancreatic cancer growth by suppressing Wnt/β-catenin/TCF-LEF1 pathway.

### Riluzole inhibited the expression of TCF-LEF1 target genes

**Figure 4:**
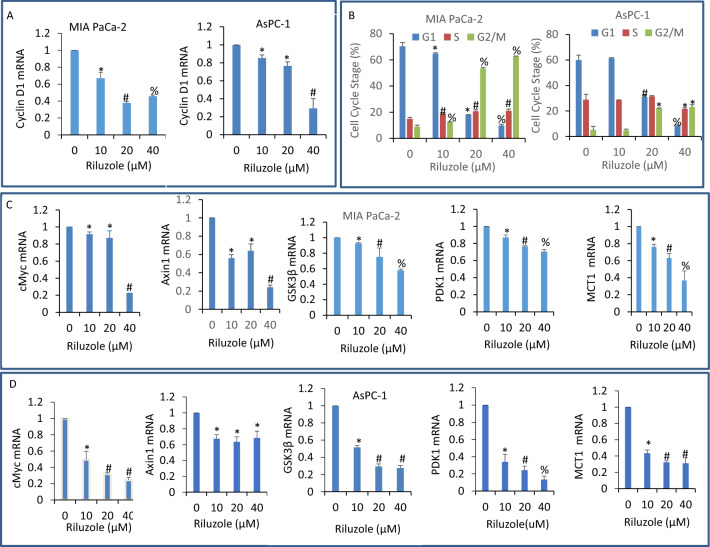
Inhibition of TCF-LEF1 target genes and induction of growth arrest at G2/M stage by riluzole. (**A**), Pancreatic cancer MIA PaCa-2 and AsPC-1 cells were treated with riluzole (0–40 µM) for 36 h. RNA was isolated and the expression of cyclin D1 was measured by q-RT-PCR. Data represent mean ± SD (n = 4). *, # and % = significantly different from control and each other; *p* < 0.05. (**B**), Cell cycle analyses. MIA PaCa-2 and AsPC-1 cells were treated with riluzole (0–40 µM) for 48 h. Cells were harvested, fixed, stained with propidium iodide (PI) and analysed with flow cytometer. *, # and % = significantly different from control and each other; *p* < 0.05. (**C**), MIA PaCa-2 cells were treated with riluzole (0–40 µM) for 36 h. RNA was isolated and the expression of cMyc, Axin1, GSK3β, PDK1, and MCT1 was measured by q-RT-PCR. Data represent mean ± SD (n = 4). *, # and % = significantly different from control and each other; *p* < 0.05. (**D**), AsPC-1 cells were treated with riluzole (0–40 µM) for 36 h. RNA was isolated and the expression of cMyc, Axin1, GSK3β, PDK1, and MCT1 was measured by q-RT-PCR. Data represent mean ± SD (n = 4). *, # and % = significantly different from control and each other; *p* < 0.05.

The Wnt/β-catenin pathway is dysregulated in pancreatic ductal adenocarcinoma^47,48^. Although the activation of this pathway is an important component of normal development, its aberrant activation leads to a more aggressive phenotypes, suggesting it can be targeted for the treatment of pancreatic cancer. Since riluzole inhibited Wnt/β-catenin/TCF-LEF1 pathway, we next sought to examine the effects of riluzole on downstream targets of Wnt pathway. These downstream targets regulate cell cycle, cell growth, components of Wnt pathway, and metabolism. Riluzole inhibited the expression of cyclin D1 in both MIA PaCa-2 and AsPC-1 cells (Fig. 4A). Riluzole treatment of MIA PaCa-2 cells resulted in an inhibition of G1 stage and an increase in G2/M stage of cell cycle (Fig. 4B). Riluzole treatment also caused a slight but significant increase in S phase of cell cycle in MIA PaCa-2 cells. By comparison, 10 μM dose of riluzole has no significant effect on cell cycle in AsPC-1 cells (Fig. 4B). Higher doses of riluzole (20 and 40 μM) inhibited G1 stage and increased G2/M stage of cell cycle. A slight inhibition in S stage was seen with the highest dose (40 μM) of riluzole in AsPC-1 cells.

Transcription factor TCF/LEF induces the expression of cMyc, Axin1, GSK3B, pyruvate dehydrogenase kinase 1 (PDK1), and monocarboxylate lactate transporter 1 (MCT-1). Riluzole inhibited the expression of cMyc, Axin1, GSK3B, PDK1, and MCT1 in both MIA PaCa-2 and AsPC-1 cells (Fig. 4C,D). These data suggest that riluzole can regulate cell cycle, cell proliferation, and glucose metabolism in pancreatic cancer cells by modulating the expression of components of Wnt pathway and its down-stream targets.

### Riluzole inhibited Bcl-2 and induced Bax expression and disrupted mitochondrial membrane potential in pancreatic cancer cells

**Figure 5:**
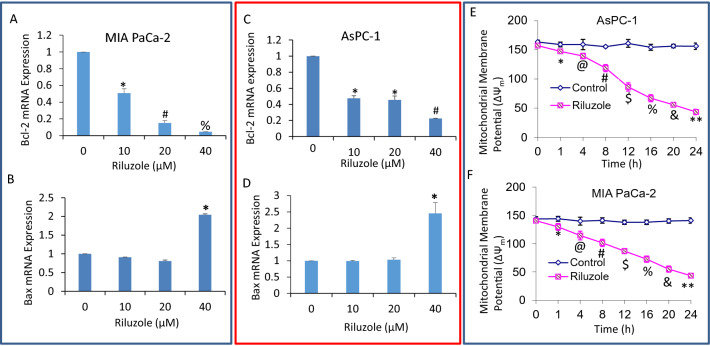
Inhibition of Bcl-2, induction of Bax and drop in mitochondrial membrane potential by riluzole. (**A–D**), MIA PaCa-2 and AsPC-1 cells were treated with riluzole (0–40 µM) for 36 h. RNA was isolated and the expression of Bcl-2 and Bax was measured by q-RT-PCR. Data represent mean ± SD (n = 4). *, #, and % = significantly different from control and each other; *p* < 0.05. (**E,F**), AsPC-1 and MIA PaCa-2 cells were treated with or without riluzole (20 µM) for 6 h, and mitochondrial membrane potential was measured as described in Materials and methods. Data represent mean ± SD (n = 4). *, @, #, $, %, & and ** = significantly different from control and each other; *p* < 0.05.

Bcl-2 family members play a major role in regulating mitochondrial functions including mitochondrial membrane potential. We, therefore, measured the expression of antiapoptotic Bcl-2, proapoptotic Bax and mitochondrial membrane potential. Riluzole inhibited Bcl-2 and induced Bax expression in pancreatic cancer cells (Fig. 5A–D). Since Bcl-2 and Bax act at the level of mitochondria and regulate permeability transition, we measured the effects of riluzole on mitochondrial membrane potential. Treatment of AsPC-1 and MIA PaCa-2 cells with riluzole caused a drop in mitochondrial membrane potential in a dose-dependent manner (Fig. 5E,F). These data suggest the involvement of mitochondria in riluzole-induced apoptosis through regulation of Bcl-2 and Bax.

### Riluzole inhibited glucose transporter 2 (Glut2) expression, glucose uptake, lactate dehydrogenase A (LDHA-A) expression, and nicotinamide adenine dinucleotide (NAD +) level

**Figure 6:**
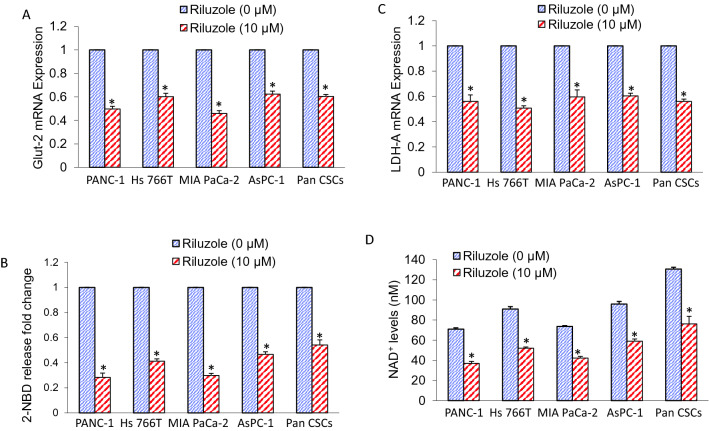
Riluzole inhibits Glut2 expression, glucose uptake, LDHA-A expression, and NAD^+^ level. (**A**), Pancreatic cancer (PANC-1, Hs 766 T, MIA PaCa-2 and AsPC-1) cells and Pan CSCs were treated with riluzole (0–10 µM) for 36 h. RNA was extracted and the expression of Glut-2 was measured by q-RT-PCR. Data represent mean ± SD (n = 4). * = significantly different from control; *p* < 0.05. (**B**), Glucose uptake. Pancreatic cancer cells and CSCs were labelled with 2.5 µg/ml of 2-deoxy-2-[(7-nitro-2,1,3-benzoxadiazol-4-yl)amino]-D-glucose (2-NBD Glucose) for 30 min and treated with riluzole for 24 h. Cells were washed and resuspended in PBS. Fluorescence was measured (excitation 544 nm and emission at 590 nm). Data represent mean ± SD (n = 4). * = significantly different from control; P < 0.05. (**C**), Pancreatic cancer **(**PANC-1, Hs 766 T, MIA PaCa-2 and AsPC-1) cells, and Pan CSCs were treated with riluzole (0–10 µM) for 36 h. RNA was isolated and the expression of LDH-A was measured by q-RT-PCR. Data represent mean ± SD (n = 4). * = significantly different from control; *p* < 0.05. (**D**), Pancreatic cancer **(**PANC-1, Hs 766 T, MIA PaCa-2 and AsPC-1) cells were treated with riluzole (0–10 µM) for 1 h and total cellular NAD + concentration was measured at 450 nm by a NAD/NADH assay kit (Cayman). Data represent mean ± SD (n = 4). * = significantly different from control; *p* < 0.05.

The dysregulated canonical WNT/β-catenin pathway can modify metabolic enzymes^49–51^. Since cancer cells and CSCs are addicted to aerobic glycolysis and lactate (Warburg effect), we measured the expression of glucose transporter 2 (Glut2), glucose uptake, lactate dehydrogenase-A (LDH-A) expression, and nicotinamide adenine dinucleotide (NAD +) level in PANC-1, Hs 766 T, MIA PaCa-2, AsPC-1 and Pan CSCs. Riluzole inhibited Glut2 expression and glucose uptake by pancreatic cancer cells and CSCs (Fig. 6A,B). Furthermore, riluzole inhibited LDH-A expression and NAD + level in pancreatic cancer cells and CSCs (Fig. 6C,D). Overall, these data suggest that riluzole can inhibit glucose uptake and lactate production by inhibiting Glut2 and LDH-A expression, respectively.

### Glutamine is required for pancreatic cancer

**Figure 7:**
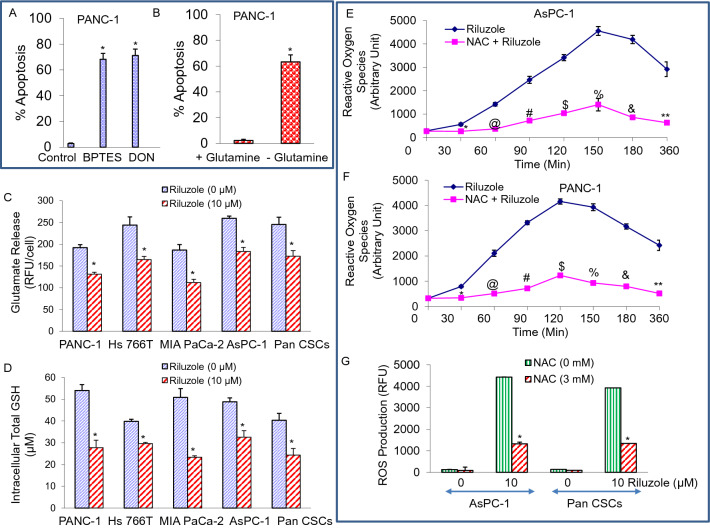
Requirement of glutamine, inhibition of glutamate efflux and GSH level, and upregulation of reactive oxygen species (ROS) in PDAC. (**A**), PANC1 cells were grown in the absence or presence of glutaminase (GLS) inhibitor (BPTES or DON, 5 mM) with glutamine (2 mM) in the medium. After 48 h, apoptosis was measured by TUNEL assay. Data represent mean ± SD (n = 4). * = significantly different from control, *p* < 0.05. (**B**), PANC-1 cells were grown in the presence (2 mM) or absence of glutamine for 48 h. Apoptosis was measured by TUNEL assay. Data represent mean ± SD (n = 4). * = significantly different from control, *p* < 0.05. (**C**), PANC-1, Hs 766 T, MIA PaCa-2, AsPC-1, and Pan CSCs were treated with riluzole (0–10 µM) for 24 h. Glutamate release was measured by the Amplex Red Glutamic Acid/Glutamate Oxidase Assay Kit with a fluorometer using excitation at 540 nm and emission at 590 nm (Invitrogen). Data represent mean ± SD (n = 4). * = significantly different from control, *p* < 0.05. (**D**), Pancreatic cancer PANC-1, Hs 7ssT, MIA PaCa-2, AsPC-1 cells and CSCs were treated with riluzole (0–10 µM) for 24 h. Intracellular total GSH was detected by measuring the product of glutathionylated DTNB at 405 nm (GSH assay kit, Cayman Chemical). Data represent mean ± SD (n = 4). * = significantly different from control, P < 0.05. (**E** and **F**), AsPC-1 and PANC-1 cells were pre-treated with NAC (3 mM) for 2 h, followed by treatment with riluzole (0–10 µM) for 24 h. Cells were labelled with 2’,7’-dichlorofluorescein diacetate (DCFDA / H2DCFDA) and ROS production was measured for various time points (0- 360 min) with a fluorometer using excitation at 495 nm and emission at 529 nm (Cellular Reactive Oxygen Species Detection Assay Kit, Abcam). Data represent mean ± SD (n = 4). *, @, #, $. %, &, and ** = significantly different from control; *p* < 0.05. (**G**), AsPC-1 and Pan CSCs were pre-treated with NAC (3 mM) for 2 h, followed by treatment with riluzole (0–10 µM) for 24 h. Cells were labelled with 2’,7’-dichlorofluorescein diacetate (DCFDA / H2DCFDA) and ROS production was measured at 120 min with a fluorometer using excitation at 495 nm and emission at 529 nm. Data represent mean ± SD (n = 4). * = significantly different from control and each other; *p* < 0.05.

Pancreatic cancer cells are addicted to amino acid glutamine to fuel anabolic processes^34,52^. In order to test the requirement of the glutamine for PDAC, we have grown PANC-1 cells in the glutamine free medium and also in the presence of glutaminase (GLS) inhibitors [(bis-2-(5-phenylacetamido-1,2,4-thiadiazol-2-yl) ethyl sulphide (BPTES) or 6-diazo-5-oxo-L-norleucin (DON)]. The data demonstrate that inhibition of GLS by BPTES or DON induced apoptosis in PANC-1 cells (Fig. 7A,B). Similarly, glutamine deprivation induced apoptosis in PANC1 cells. GLS inhibitors induced apoptosis to the same extent as glutamine deprivation. These data suggest that glutamine is required for PDAC.

### Riluzole inhibited glutamate release, and glutathione (GSH) level, and elevated reactive oxygen species (ROS) in pancreatic cancer cells and CSCs

Since riluzole regulates glutamate metabolisms in ALS^18,53^, we, therefore, sought to examine the effects of riluzole on glutamate release, intracellular GSH level, and ROS production. Riluzole inhibited glutamate release in pancreatic cancer cells (PANC-1, Hs 766 T, MIA PaCa-2, AsPC-1) and Pan CSCs (Fig. 7C). Since glutathione (GSH) participates in oxidative stress response and defends against cellular toxicity, we sought to examine the effects of riluzole on intracellular GSH and ROS production. Riluzole inhibited intracellular GSH level in pancreatic cancer cells and CSCs (Fig. 7D). Treatment of AsPC-1 and PANC-1 cells with riluzole resulted in elevation of ROS reaching a plateau at 150 and 120 min, respectively (Fig. 7E,F). Pre-treatment of cells with N-acetyl-L-cysteine (NAC), an antioxidant, inhibited riluzole-induced ROS production (Fig. 7E–G). These data suggest that riluzole can increase intracellular glutamate, inhibit GSH and elevate ROS, which may be one of the mechanisms of apoptosis induction through mitochondrial dysfunction.

### Riluzole inhibited pancreatic cancer development in KPC (Pdx1-Cre, LSL-Trp53^R172H^, and LSL-Kras^G12D^) mice

**Figure 8:**
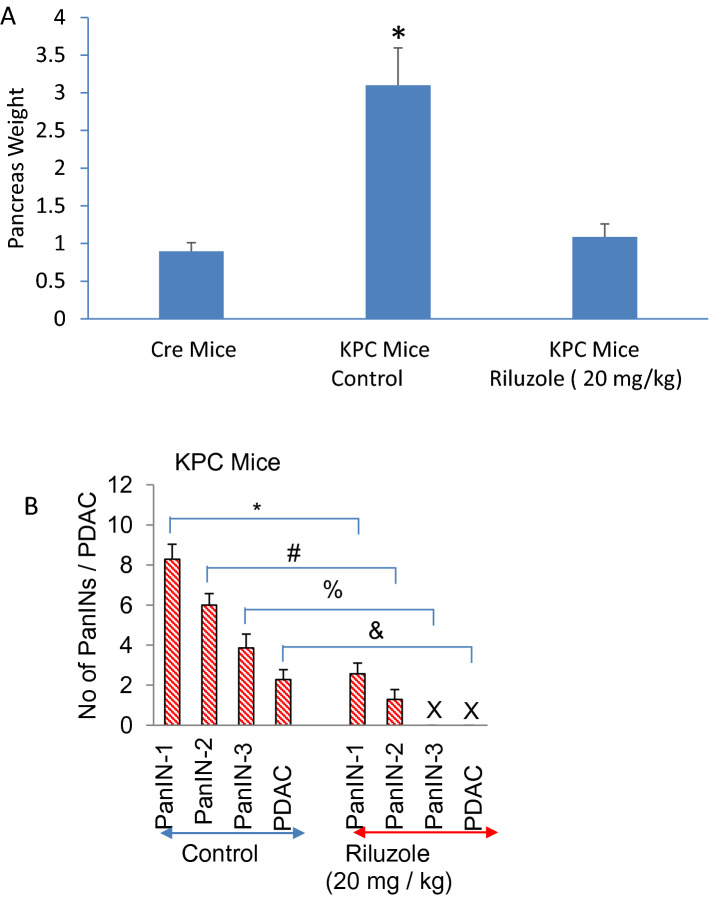
Riluzole inhibits pancreatic cancer growth in KPC (Pdx1-Cre, LSL-Trp53^R172H^, and LSL-Kras^G12D^) transgenic mice. KPC mice (about 6 weeks old) were injected ip with riluzole (20 mg/kg, Monday through Friday) for 12 weeks. At the end of the treatment, mice were sacrificed. (**A**) Pancreas Weight. Mice were euthanized and pancreas weight was taken from Cre mice (normal, untreated), KPC mice (control, untreated), and KPC mice (treated with riluzole 20 mg/kg). Data represent mean ± SD (n = 7). * and # = significantly different from control group; *p* < 0.05. (**B**), Histological examination of the pancreas was performed by H&E staining. Numbers of PanINs and PDAC were quantified. Data represent mean ± SD (n = 7). *, #, %, and & = significantly different from control group; *p* < 0.05. X = not detected.

KPC mice mimic pancreatic cancer development in humans and they have been used to test the efficacy of new drugs for the treatment of pancreatic cancer^44,54^. Since riluzole inhibited cell proliferation and colony formation, and induced apoptosis in vitro, we next sought to examine the effects of riluzole on pancreatic cancer growth and development in KPC (Pdx1-Cre, LSL-Trp53^R172H^, and LSL-Kras^G12D^) mice by treating them for three months. Treatment of KPC mice with riluzole inhibited pancreas weight, which was similar to that of untreated Cre mice (Fig. 8A). Control group of KPC mice developed PanIN 1–3 lesions and PDAC at about 4.5 months (Fig. 8B). By comparison, numbers of PanIN 1 and 2 lesions were significantly inhibited in the riluzole-treated group. Interesting, PanIN3 and PDAC were not observed in the riluzole-treated group. Riluzole inhibited pancreatic cancer growth and development in KPC mice. Overall, our transgenic mice data suggest that riluzole is effective in inhibiting pancreatic cancer growth and development in mice, and can be used for the treatment of pancreatic cancer.

## Discussion

We have shown for the first time that riluzole inhibits pancreatic cancer growth and development by regulating glucose and glutamine metabolisms. The existence of cancer stem cells (CSCs) in the pancreas hinders the development of new drugs because CSCs can contribute towards therapy failure and drug resistance. Riluzole not only inhibits growth of cancer cells but also cancer stem cells which are generally responsible for drug resistance and chemotherapy failure. Furthermore, riluzole affects cancer cell metabolisms and mitochondrial homeostasis which can modify responses to therapy. Since Wnt/β-catenin/TCF-LEF pathway is highly activated in pancreatic cancer, inhibition of Wnt/β-catenin/TCF-LEF pathway by riluzole will not only inhibit cancer cell proliferation but also regulate those genes which play major roles in cancer cell metabolisms.

In the present study, anticancer activities of riluzole were observed in pancreatic cancer cells which show genetic variability in Kras and p53 status. Riluzole was also effective in pancreatic CSCs which generally do not respond to anticancer drugs and are responsible for drug resistance and cancer relapse. In addition to apoptosis, riluzole treatment also caused growth arrest at G2/M stage and reduced G1 stage of cell cycle which was accompanied by inhibition of cyclin D1 expression. Similarly, another study has demonstrated that riluzole inhibited cell viability and colony formation, blocked cell cycle, and induced apoptosis in pancreatic cancer cells^55^. Furthermore, in other studies riluzole exerted anti-tumor activities in breast cancer cells independent of metabotropic glutamate receptor-1, and inducing mitotic arrest^56,57^. Interestingly, riluzole was not effective in human normal pancreatic ductal epithelial cells, suggesting its activity was limited to malignant cells and thus offers hope for the treatment of pancreatic cancer.

Mitochondria glutamine metabolism plays an essential role in maintaining mitochondrial functions and regulates cellular sensitivity to DNA damage^11^. Glutamine oxidizes in mitochondria and produces ATP. Pancreatic cancer cells are addicted to glutamate for survival^58–60^. Riluzole inhibits glutamate release through inactivation of voltage-dependent ion channels^19–21^. In prostate cancer, serum glutamate levels directly correlate with Gleason score and glutamate blockade decreases proliferation, migration, and invasion and induces cell death^61^. Blocking glutamate release by riluzole inhibits cell proliferation in glioblastoma, melanoma, breast and prostate cancer^19,56,62–64^. Since riluzole inhibited glutamate release and GSH level, and increased ROS production in pancreatic cancer cells and CSCs, this could be considered as one of the mechanisms of apoptosis induction.

Upregulation of WNT/β-catenin pathway induces aerobic glycolysis (known as Warburg effect), through activation of GLUT, PDK1, pyruvate kinase M2 (PKM2), MCT-1, LDH-A and inactivation of pyruvate dehydrogenase complex. Oncogenic Myc regulates the expression of glycolysis genes, such as PDK1, GLUT1, HK2, and LDH-A^65,66^. WNT/β-catenin pathway directly regulates Myc expression. The aerobic glycolysis supplies a large part of glucose into lactate regardless of oxygen. Aerobic glycolysis is less efficient in producing ATP compared to oxidative phosphorylation. Phosphorylation of PDK-1 inhibits the PDH, and a large part of pyruvate cannot be converted into acetyl-CoA in mitochondria and only a part of acetyl-CoA can enter the TCA cycle. Cytosolic pyruvate is converted into lactate through the enzymatic activity of LDH-A. In the present study, riluzole inhibited c-Myc and GLUT-2 expression, glucose uptake, LDH-A expression, and NAD^+^ levels. Furthermore, knockdown of β-catenin results in reduced glutamate transporter (GLT-1) and glutamine synthetase (GS) expression in astrocytes^35^. Similar to the action of riluzole, inhibition of mitochondrial ATP production downregulated Wnt/beta-catenin signaling pathway^67,68^.

Bcl-2 family members regulate cell growth, survival, and apoptosis^69–72^. Mainly, anti-apoptotic members such as Bcl-2 and Bcl-X_L_, enhance cell growth and proliferation, whereas pro-apoptotic members such as Bax and Bad, induce apoptosis. Following cellular stress, Bak and/or Bax are activated and compromise the integrity of the outer mitochondrial membrane (OMM) resulting in permeabilization of mitochondrial outer membrane. As a result of MOMP, pro-apoptotic proteins (*e.g.,* cytochrome c) move to the cytoplasm where they activate caspase to induce apoptosis. Anti-apoptotic Bcl-2 family proteins regulate cellular survival by tightly controlling the interactions between Bak/Bax and the BH3-only proteins capable of directly inducing Bak/Bax activation. In the present study, riluzole inhibited the expression of Bcl-2 and induced the expression of Bax and caused a drop in mitochondrial membrane potential leading to induction of apoptosis. These data suggest that riluzole can act at the level of mitochondrial to regulate apoptosis.

In conclusion, the proapoptotic and antiproliferative effects of riluzole are exerted through rewiring of mitochondrial signals that are specific to pancreatic cancer cells. Glutamine, a mitochondrial substrate, could be required for maintenance of mitochondrial membrane potential and integrity and for support of the NADPH production needed for redox control and macromolecular synthesis. Furthermore, metabolic reprogramming of pancreatic cancer mediated by glutamate inhibition elicits unique vulnerabilities to malignant cells, but not to normal pancreatic epithelial cells. Our in vitro and in vivo data suggest that riluzole can be used for the treatment of pancreatic cancer.

## Supplementary Information

Supplementary Information 1.

Supplementary Information 2.

Supplementary Information 3.

Supplementary Information 4.

Supplementary Information 5.

Supplementary Information 6.

## Data Availability

The data that support the findings of this study are available from the corresponding author upon reasonable request.
