# Supplementary figures and images for: Riluzole regulates pancreatic cancer cell metabolism by suppressing the Wnt-β-catenin pathway
Source: Sci Rep. 2022 Jun 30;12:11062. doi: 10.1038/s41598-022-13472-y (PMC9246955; doi:10.1038/s41598-022-13472-y)

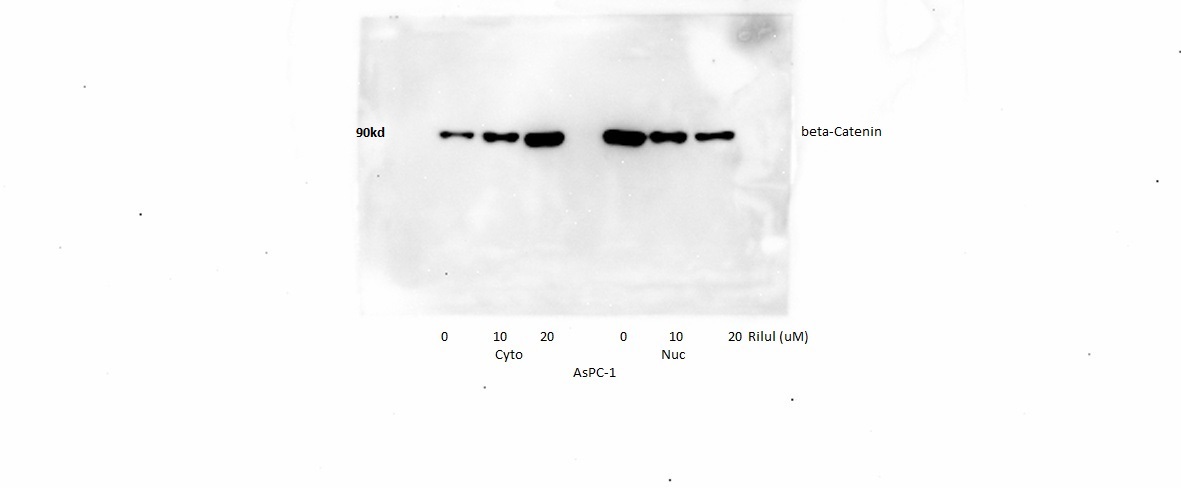

Supplement: Supplementary file 1 — Supplementary Information 1. [file 41598_2022_13472_MOESM1_ESM.jpg]

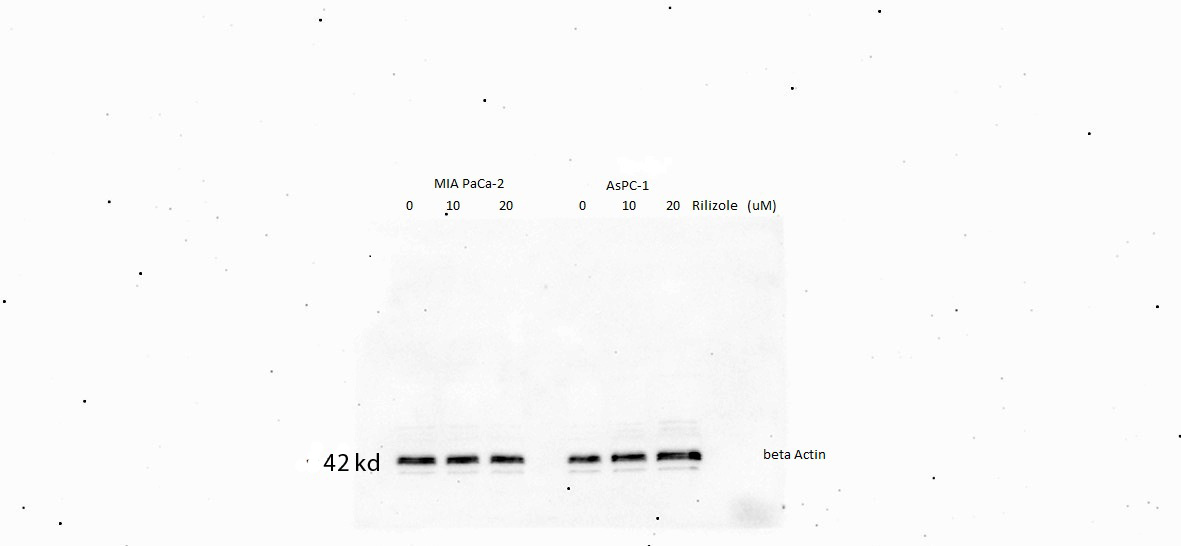

Supplement: Supplementary file 3 — Supplementary Information 3. [file 41598_2022_13472_MOESM3_ESM.jpg]

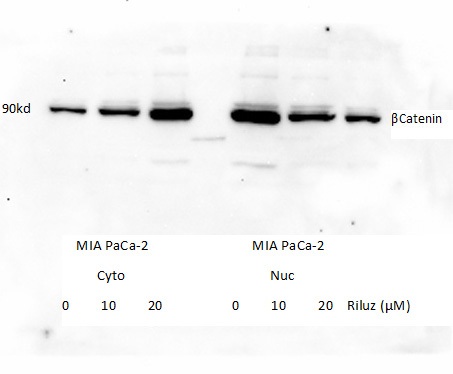

Supplement: Supplementary file 4 — Supplementary Information 4. [file 41598_2022_13472_MOESM4_ESM.jpg]

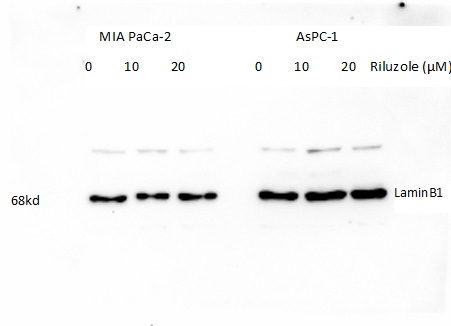

Supplement: Supplementary file 5 — Supplementary Information 5. [file 41598_2022_13472_MOESM5_ESM.jpg]
