# Supplementary material for: Riluzole regulates pancreatic cancer cell metabolism by suppressing the Wnt-β-catenin pathway
Source: Sci Rep. 2022 Jun 30;12:11062. doi: 10.1038/s41598-022-13472-y (PMC9246955; doi:10.1038/s41598-022-13472-y)
Supplement: Supplementary file 6 — Supplementary Information 6. [file 41598_2022_13472_MOESM6_ESM.doc]

**Supplemental File**

**Figure Legend**

1. Gel 1.1: Western blot of beta-actin of MIA and AsPC-1 cells treated with Riluzole.
2. Gel 2: Western blot of beta-catenin (cytoplasmic and nuclear fraction) of

AsPC-1 cells treated with Riluzole.

1. Gel 3: Western blot of beta-catenin (cytoplasmic and nuclear fraction) of MIA cells treated with Riluzole.
2. Gel 4: Western blot of Lamin B1 (nuclear fraction) of MIA and AsPC-1 cells treated with Riluzole.
